# Relationship between vestibular loss and the risk of dementia using the 2002–2019 national insurance service survey in South Korea

**DOI:** 10.1038/s41598-023-42598-w

**Published:** 2023-10-05

**Authors:** Sung Jin Lim, Serhim Son, Younghan Chung, Sang Yeop Kim, Hangseok Choi, June Choi

**Affiliations:** 1 Department of Otorhinolaryngology-Head and Neck Surgery, Ansan Hospital, Korea University College of Medicine, 123 Jeokgeum-ro, Danwon-gu, Ansan-si, Gyeonggi-do 15355 Republic of Korea; 2 Department of Biostatistics, Korea University College of Medicine, Seoul, Republic of Korea; 3 Medical Science Research Center, Korea University College of Medicine, 73, Goryeodae-ro, Seongbuk-gu, Seoul, 02841 Republic of Korea; 4 Department of Medical Informatics, Korea University College of Medicine, Seoul, Republic of Korea

**Keywords:** Diseases, Medical research

## Abstract

This retrospective cross-sectional study assessed older adults aged between 40 and 80 years, registered in the Korean National Health Insurance Service database from 2002 to 2019 to investigate the association between vestibular loss and the risk of dementia. The population was divided into three groups (general, vestibular loss, and hearing loss). The hazard ratios (HRs) of dementia in the vestibular and hearing loss groups were calculated using national population data. In total, 2,347,610 individuals were identified (general: 2,145,609, vestibular loss: 60,525, hearing loss: 141,476). Mean ages were 53.29 years, 58.26 years, and 58.52 years, respectively. Dementia occurred in 127,081 (IR = 4.91 per 1000 person-years), 7705 (IR = 10.79 per 1000 person-years), and 16,116 (IR = 9.63 per 1000 person-years) patients. The vestibular and hearing loss groups had hazard ratios for dementia of 1.084 (95% CI, 1.059–1.110) and 1.074 (95% CI, 1.056–1.092), respectively, compared with the general group. The results of the current study suggest that vestibular loss increases the risk of developing dementia. Therefore, similar to hearing loss, vestibular loss should be considered a risk factor for dementia, and treatments such as adequate vestibular rehabilitation may reduce this risk.

## Introduction

The vestibular system plays a primary role in sensing head movement and orientation in space. This system projects to numerous parts of the central nervous system, including the cerebellum, brainstem, and cortical areas. Dementia is a broad term referring to severe impairment of memory, language, problem-solving, and other cognitive abilities disrupting daily life. As age demographics shift, dementia constitutes a major public health, social, and economic challenge worldwide and is expected to affect approximately 130 million people by 2050^1^. Due to its impact, numerous studies have been conducted to reveal its pathophysiology, and a growing body of literature suggests a potential link between vestibular loss and increased dementia risk^2–5^.

Previous studies have suggested that the vestibular system is closely related to cognitive functions, especially visuospatial processing^2,6^.

Animal studies have demonstrated that vestibular inputs play a role in spatial memory and navigation. Rat studies have shown that bilateral vestibular lesions result in the aberrant activity of the rats’ place cells, which is thought to sustain the brain’s cognitive map of space in the hippocampus^7–11^. Another study compared rats with bilateral vestibular lesions to sham animals (preserved vestibular function but removed tympanic membrane). The sham animals had their tympanic membrane removed as rats with bilateral vestibular lesions had the possibility of cochlear injury. The study demonstrated that sham animals performed better on spatial tasks than animals with vestibular lesions^8–11^. This suggests that vestibular function is more important than auditory function for spatial memory.

The results of human studies correlated with those of animal studies. Bilateral vestibulopathy is associated with impairment of spatial cognition, immediate memory, processing speed, and executive function^4,12,13^. Other studies have documented that patients with vestibular disorders show impaired spatial memory and navigation, as well as reduced hippocampal volumes^5,14^. In addition, there is a higher prevalence of vestibulopathy among people with cognitive loss^3^.

To our knowledge, no studies have been published on this topic in large-scale populations. This study aimed to investigate the association between vestibular loss and risk of dementia using a large-scale database. In South Korea, the national population is registered with the National Health Insurance Service, and all medical data are organized in the National Health Information Database. Utilizing nationwide data might provide a deeper understanding of this association.

## Results

Our study included a total of 2,347,610 participants. Of these, 2,145,609 participants were classified into the general control group (those not seeking clinic visits for vestibular or hearing loss), 60,525 participants made up the vestibular loss group, and 141,476 participants were in the hearing loss group. The mean age for each group was 53.29 ± 10.43 years, 58.26 ± 10.76 years, and 58.52 ± 10.91 years, respectively. The other demographic characteristics and detailed information are presented in Table 1.

**Table 1:** Demographic characteristics of the study population.

	General (n = 2,145,609)	Only VL (n = 60,525)	Only HL (n = 141,476)	*P* value
Age, mean (SD)	53.29 (10.43)	58.26 (10.76)	58.52 (10.91)	< 0.001
< 65, n (%)	1,758,551 (81.96)	40,715 (67.27)	93,920 (66.39)	
≥ 65, n (%)	387,058 (18.04)	19,810 (32,73)	47,556 (33.61)	
Income (bottom 20%), n (%)	385,300 (18.46)	10,942 (18.36)	24,272 (17.43)	< 0.001
Sex, n (%)				< 0.001
Male	1,126,958 (52.52)	17,221 (28.45)	71,204 (50.33)	
Female	1,018,651 (47.48)	43,304 (71.55)	70,272 (49.67)	
Diabetes, n (%)	179,618 (8.37)	7,827 (12.93)	19,392 (13.71)	< 0.001
Hypertension, n (%)	383,160 (17.86)	22,106 (36.52)	40,717 (28.78)	< 0.001
Dyslipidemia, n (%)	62,801 (2.93)	4156 (6.87)	7530 (5.32)	< 0.001
Ischemic heart disease, n (%)	66,274 (3.09)	4597 (7.6)	8699 (6.15)	< 0.001
Stroke, n (%)	43,313 (2.02)	5045 (8.34)	5088 (3.6)	< 0.001
Cancer, n (%)	127,459 (5.94)	5513 (9.11)	12,571 (8.89)	< 0.001

*VL* vestibular loss; *HL* hearing loss.

*P*-values were calculated using chi-square test.

**Figure 1:**
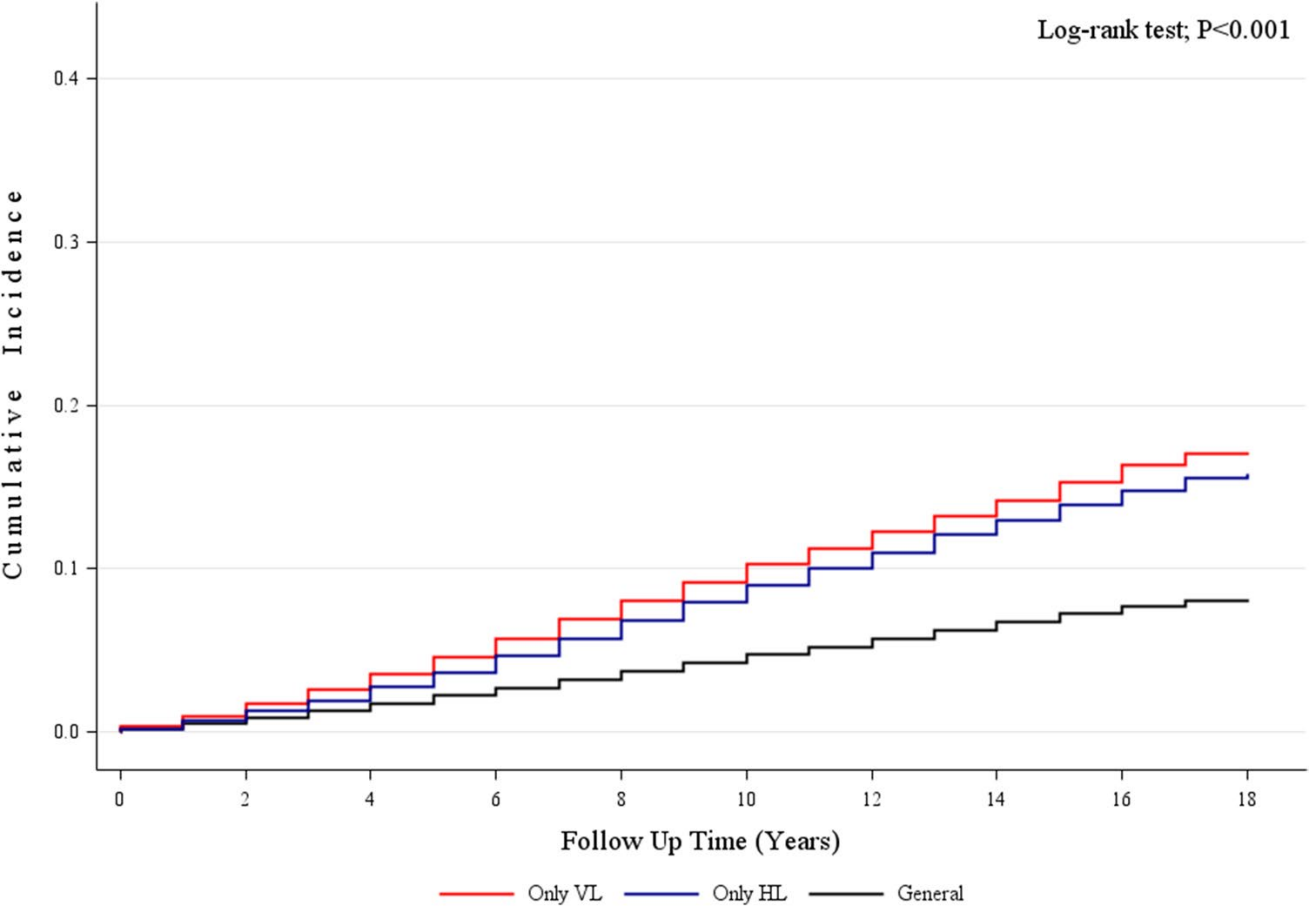
Incidence rate according to vestibular loss and hearing loss. Abbreviations: VL, vestibular loss; HL, hearing loss.

**Figure 2:**
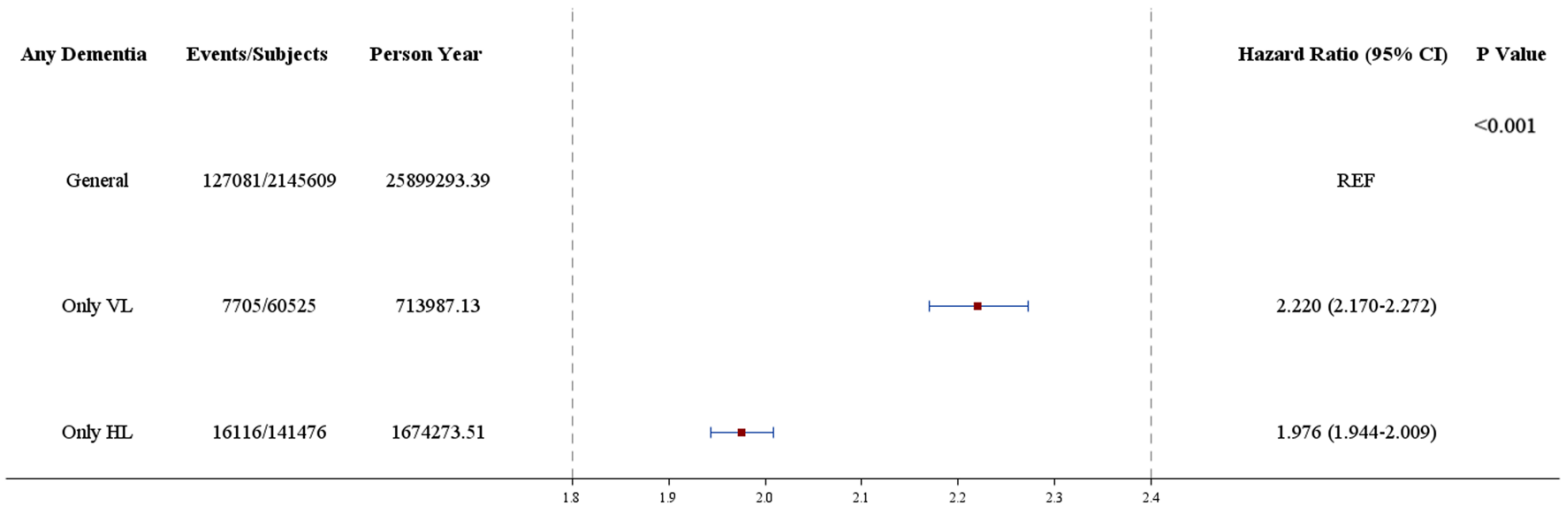
Hazard ratio of dementia in vestibular and hearing loss groups.

The incidence rates of dementia in each group were 127,081 [5.9%], 7705 [12.7%], and 16,116 [11.4%] patients, respectively. The Kaplan–Meier analysis, revealed a higher IR of dementia in the vestibular and hearing loss groups compared to the control group (log-rank test, *P* < 0.001) (Fig. 1). During the follow-up period, 7705 individuals in the vestibular loss group (IR = 10.79 per 1000 person-years) and 16,116 in the hearing loss group (IR = 9.63 per 1000 person-years) were diagnosed with dementia. In contrast, 127,081 individuals in the general control group were diagnosed with dementia (IR = 4.91 per 1000 person-years). The univariate Cox proportional hazards model yielded an HR for dementia of 2.22 (95% CI, 2.170–2.272; *P* < 0.001) in the vestibular loss group and 1.976 (95% CI, 1.944–2.009; *P* < 0.001) in the hearing loss group (Fig. 2). After adjusting for potential confounding variables, including age, sex, and underlying disease, the HR for dementia was 1.084 (95%CI, 1.059–1.110; *P* < 0.001) in the vestibular loss group and 1.074 (95%CI, 1.074–1.092; *P* < 0.001) in the hearing loss group (Table 2).

**Table 2:** Adjusted hazard ratios of dementia in vestibular and hearing loss groups.

	Number	Dementia	IR (per 1000)	HR (95% CI)	*P* value
General	2,145,609	127,081	4.91	1 (ref.)	< 0.001
Only VL	60,525	7,705	10.79	1.084 (1.059, 1.110)	
Only HL	141,476	16,116	9.63	1.074 (1.056, 1.092)	

*VL* vestibular loss; *HL* hearing loss; *IR* incidence ratio; *HR* hazard ratio.

HR: adjusted for age, sex, income, diabetes, hypertension, dyslipidemia, ischemic heart disease, stroke, and cancer.

*P*-values were calculated using cox’s proportional model.

## Discussion

This study aimed to explore the association between vestibular loss and risk of dementia using a large-scale database. To our knowledge, no studies have been published on this topic in large-scale populations.

In South Korea, all citizens are enrolled in the KNHIS, and all medical data is well-organized. Given the detailed medical practices, fee-for-service information, and prescriptions that the KNHIS data provides, it is frequently used in medical studies.

Recently, several nationwide studies have documented their databases on dementia registration^15–17^. Although each study had slightly varies in the ICD-10 codes for dementia and features for defining dementia, such as medications to treat dementia, underlying or contributing causes of death, and hospital admissions, our definition aligns with previous studies^15–19^.

Our findings suggest that vestibular loss increased the risk of dementia, consistent with the previous studies^2,20–23^. A previous study showed that older adults with bilateral vestibulopathy had clinically significant lower scores than those without on the Repeatable Battery for the Assessment of Neuropsychological Status for Hearing-Impaired Individuals (RBANS-H), designed to assess cognitive function in individuals with hearing loss^24^. Additionally, another study found that patients with bilateral saccular and semicircular canal vestibular loss had lower scores on the Trail Making Test (TMT) and the Benton Visual Retention Test Part-C (BVRT-C) than the control group, indicating a link between bilateral semicircular canal and saccular vestibular loss and loss of various domains of cognition^23^. Regarding brain volume, patients with chronic bilateral vestibular loss had a 16.9% decrease in hippocampal volume, a region crucial to various aspects of memory processing^12^. Conversely, the prevalence of vestibular loss was higher among individuals with cognitive loss^3^.

Even though accumulating evidence supports an association between vestibular and cognitive loss, a causal relationship has not yet been validated. Several hypotheses, particularly regarding the association between vestibular loss and Alzheimer's disease, have been suggested. First, vestibular loss reduces the vestibular input to the brain, leading to brain atrophy. A previous study reinforced this hypothesis by demonstrating decreased hippocampal atrophy in individuals with vestibular loss^12^. Second, people with vestibular loss often fear falling and feel unsafe, limiting their participation in activities and trips. Consequently, they live a more socially isolated life^25,26^. Third, the common-cause hypothesis proposes that both vestibular and cognitive loss are the consequences of a neurodegenerative process. Several factors affect vestibular and cognitive loss, and these risk factors can coexist^22^.

**Figure 3:**
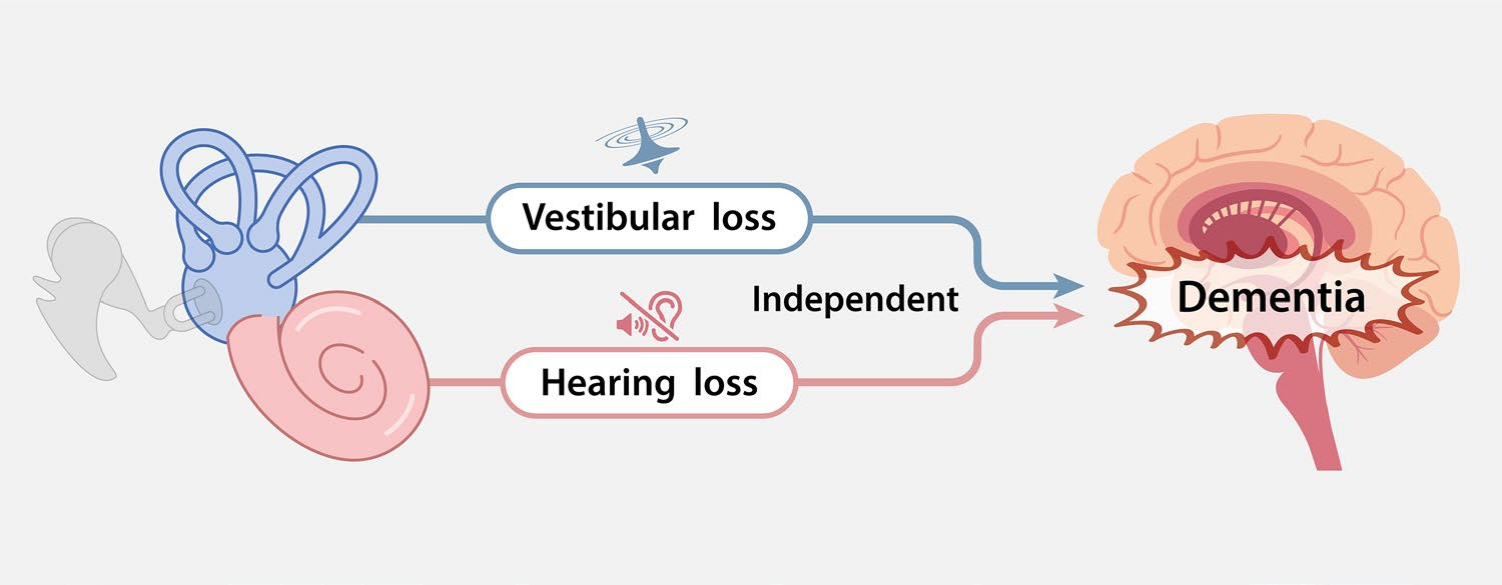
The relationship between vestibular loss and the risk of dementia.

The present study analyzed the data from over two million individuals and revealed a statistically significant difference in the incidence of dementia between the vestibular loss and general groups (Fig. 3). However, this study has some limitations. First, some participants with vestibular loss were potentially overlooked in the present study. We defined the vestibular loss group as patients who visited the clinic and were diagnosed with Benign Paroxysmal Positional Vertigo (BPPV), Meniere’s disease, or vestibular neuritis. Therefore, patients with other types of vestibular loss or those assigned by the doctor only with the dizziness code (ICD-10 code R42), were not included in this study. Second, the illness duration and symptom severity were not assessed. Third, our dataset comprised claims data collected for reimbursement purposes and were not organized for epidemiological research. Therefore, this could have introduced potential errors, such as omission or incorrect batch code assignment. Lastly, despite many studies, including ours, operating under a relatively short washout period of one year, some studies advocate for the analysis of the hazard ratio of non-acute diseases over a longer washout period of at least three years. Therefore, this study is potentially limited by the varying duration of the washout periods across different studies.

## Conclusion

This retrospective study demonstrated that vestibular loss increased the risk of dementia compared to healthy controls, with the HR of dementia being similar to that of the hearing loss group. As the prevalence of dementia is increasing, it is crucial to identify modifiable risk factors. Similar to hearing loss, vestibular loss should be considered an independent risk factor for dementia, and appropriate treatments such as adequate vestibular rehabilitation may reduce this risk. Further studies that include the duration of vestibular loss, severity of symptoms, and the impact of vestibular treatment on cognition are recommended.

## Materials and methods

### Study design and participants

Korean National Health Insurance Service (KNHIS) is a system in which all Koreans and registered foreigners must enroll^27^. KNHIS collects data such as insurance eligibility, contributions, medical history from enrolled individuals. This public data includes information about demographics such as age and sex, and medication usage such as amount, duration, and date of death^27,28^. It also provides detailed health conditions data such as cancer, dementia, and cardiovascular disease, making it the most utilized resource for medical studies. In our study, we used a customized database (DB) from the KNHIS, focusing on sensitive diagnostic codes such as F. After an internal screening process, a customized DB was provided based on the specific variables requested for the study.

**Figure 4:**
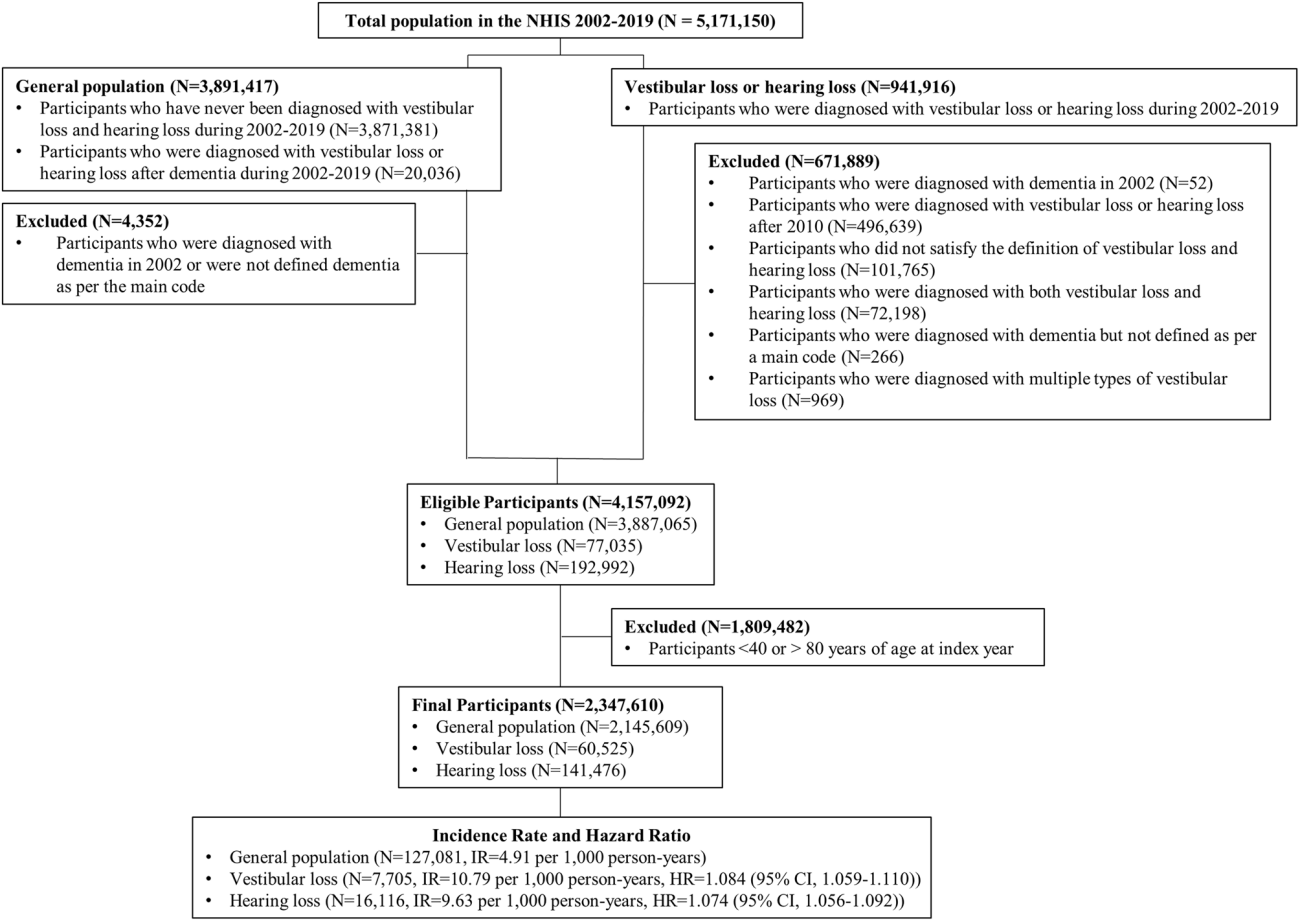
The flow chart of study participants.

For this study, we extracted the International Classification of Diseases, 10th edition codes (ICD-10) for vestibular and hearing loss. The association between hearing loss and dementia has been extensively explored, and hearing loss has been accepted as a risk factor for dementia^28–31^. For eliminating and compare the effect of hearing loss, data on the code of hearing loss were also obtained. We initially selected 5,171,150 participants registered in the KNHIS data from 2002 to 2019. Of these, we selected 3,891,417 participants who had never been diagnosed with vestibular or hearing loss or were diagnosed with these conditions after a dementia diagnosis between 2002 and 2019. After excluding 4352 participants diagnosed with dementia in 2002 or who were not diagnosed with dementia according to the principal diagnostic code, 3,887,065 participants were included in the general population. For cases of vestibular and hearing loss, we selected 941,916 participants diagnosed with either condition between 2002 and 2019. Patients meeting the following criteria were excluded: (1) diagnosed with dementia in 2002, (2) diagnosed with vestibular and hearing loss after 2010, (3) did not meet the criteria for vestibular and hearing loss, (4) diagnosed with both vestibular and hearing loss, (5) diagnosed with dementia but not according to the main code; (6) diagnosed with multiple types of vestibular loss. After excluding 671,889 participants, 77,035 participants with vestibular loss and 192,992 participants with hearing loss remained. From this population, 1,809,482 participants aged < 40 or > 80 years were excluded. Ultimately, we included 2,145,609 participants in the general study population: 60,525 with vestibular loss, and 141,476 with hearing loss. Further details are presented in Fig. 4, and the distribution of participants by index year is shown in Supplementary Table S1.

### Data collection

We compiled the data using the following variables: age (years), sex, income, and comorbidities such as diabetes, hypertension, dyslipidemia, ischemic heart disease, stroke, and cancer. Additionally, we collected the codes for Alzheimer's dementia, vascular dementia, and other causes of dementia. We included patients who were diagnosed with dementia at least twice and were prescribed medication (donepezil, rivastigmine, galantamine, and memantine) to treat the condition at least once^28,32^. In cases where more than one type of dementia was diagnosed simultaneously, we used the main code in the KNHIS data for definition. We used ICD-10 codes (Alzheimer's: F00 or G30; Vascular: F01; Others: F02, F03, or G31). Detailed definitions and diagnostic codes for vestibular loss, hearing loss, and comorbidities are provided in Supplementary Table S2.

### Statistical analysis

Data are presented as the mean ± standard deviation for continuous variables and as n (%) for categorical variables. The t-test was used to compare continuous variables and the chi-square test for categorical variables in baseline characteristics. The Kaplan–Meier survival analysis was applied to assess the cumulative incidence of dementia. Dementia incidence rates (IR) were calculated by dividing the number of dementia cases by 1000 person-years. Furthermore, Cox proportional hazard models were used to compare the risk of dementia after adjusting for demographics and comorbidities such as age, sex, income, diabetes, hypertension, dyslipidemia, ischemic heart disease, stroke, and cancer. Hazard ratios (HR) with 95% confidence intervals (CI) were calculated and a 5% significance level was considered statistically significant. Large dataset collection, exploration, and statistical analyses were conducted using SAS version 9.4 (SAS Institute, Cary, NC, USA).

### Ethical approval

This study was approved by the Institutional Review Board of the Korea University Ansan Hospital (IRB. No. 2021AS0216). The requirement for informed consent was waived by the ethics committee and the Institutional Review Board of Korea University Ansan Hospital. All methods were performed in accordance with the relevant guidelines and regulations.

### Supplementary Information

Supplementary Tables.

## Data Availability

The datasets generated and/or analyzed during the current study are not publicly available due to the legal restrictions of South Korea but are available from the corresponding author on reasonable request.
