# Supplementary material for: Relationship between vestibular loss and the risk of dementia using the 2002–2019 national insurance service survey in South Korea
Source: Sci Rep. 2023 Oct 5;13:16746. doi: 10.1038/s41598-023-42598-w (PMC10556082; doi:10.1038/s41598-023-42598-w)
Supplement: Supplementary file 1 — Supplementary Tables. [file 41598_2023_42598_MOESM1_ESM.docx]

**Supplementary Table S1** Distribution of participants by index year

| **Index year** | **General** | **Only VL** | **Only HL** |
| --- | --- | --- | --- |
| 2002 (n,%) | 269,636 (12.57%) | 5,159 (8.52%) | 13,369 (9.45%) |
| 2003 (n,%) | 255,714 (11.92%) | 5,441 (8.99%) | 14,725 (10.41%) |
| 2004 (n,%) | 256,404 (11.95%) | 6,098 (10.08%) | 15,383 (10.87%) |
| 2005 (n,%) | 258,986 (12.07%) | 6,925 (11.44%) | 17,300 (12.23%) |
| 2006 (n,%) | 265,132 (12.36%) | 8,096 (13.38%) | 19,230 (13.59%) |
| 2007 (n,%) | 271,393 (12.65%) | 9,270 (15.32%) | 19,663 (13.9%) |
| 2008 (n,%) | 280,496 (13.07%) | 9,574 (15.82%) | 20,461 (14.46%) |
| 2009 (n,%) | 287,848 (13.42%) | 9,962 (16.46%) | 21,345 (15.09%) |

VL, vestibular loss; HL, hearing loss.

**Supplementary Table S2** ICD-10 diagnostic codes

| **Variable** | **ICD-10 code** |
| --- | --- |
| **Main risk factor** |  |
| Vestibular loss | H81.1, H81.0, H81.2 |
| Hearing loss | H83.3, H90, H91 |
| **Covariates** |  |
| Diabetes | E10-E14 |
| Hypertension | I10- I15 |
| Dyslipidemia | E78.0-E78.5, E78.7-E78.9 |
| Ischemic heart disease | I20-I25 |
| Stroke | I60-I64, I69.0-I69.4 |
| Cancer | C00-C26, C30-C41, C43-C58, C60-C97, D00-D48 |
